# Transient multidomain functional improvement in advanced Alzheimer’s disease following high-dose psilocybin-containing mushroom administration: a case report

**DOI:** 10.3389/fnins.2026.1813281

**Published:** 2026-05-28

**Authors:** Marcos Lago, Mariana Cerveira, Joe Xavier Simonet

**Affiliations:** Medical Department, Associação Cruz de Ankh, São Paulo, Brazil

**Keywords:** advanced dementia, Alzheimer’s disease, functional recovery, neuroplasticity, psilocybin

## Abstract

**Background:**

Advanced Alzheimer’s disease (AD) is generally regarded as a stage of irreversible functional decline. Psilocybin is known to transiently alter large-scale brain network dynamics and to induce plasticity-related mechanisms in preclinical models, yet clinical data in advanced dementia remain lacking.

**Case presentation:**

We report the case of an octogenarian Japanese-American woman with a 10-year history of Alzheimer’s disease, including 5 years of marked hypofunction and predominantly monosyllabic speech. Baseline features included chronic urinary incontinence, executive dysfunction, dysphagia, dependent mobility, flat affect, and severe reduction in spontaneous communication. The patient received 5 g of orally administered psilocybin-containing mushrooms (Enigma strain). The acute phase was marked by autonomic activation, clinically suspected hyperthermia, profuse sweating, and a prolonged deep sleep-like state. Approximately 19 h post-administration, spontaneous autobiographical speech emerged. Over subsequent days and weeks, functional improvements included restoration of urinary continence, improved ambulation, autonomous dressing, increased emotional responsiveness, sustained social interaction, contextual memory retrieval, preserved working memory for social context, and spontaneous conversational engagement.

**Conclusion:**

This case documents transient multidomain functional improvement in advanced Alzheimer’s disease following psilocybin administration. The findings do not imply disease reversal but suggest that residual functional capacity may persist in late-stage neurodegeneration and may become transiently accessible under specific neuromodulatory conditions.

## Introduction

Advanced Alzheimer’s disease imposes profound loss of autonomy, communication, continence, mobility, and social interaction, generating severe emotional and caregiving burden. Current therapeutic strategies at this stage are largely supportive, and meaningful functional recovery is generally considered unlikely. The global burden of Alzheimer’s disease and related dementias remains substantial, with major consequences for patients, families, caregivers, and health systems (Alzheimer's Association, 2024). This clinical reality has motivated growing interest in neuromodulatory and neuroplasticity-oriented approaches capable of transiently restoring residual network function.

Psilocybin produces marked alterations in large-scale brain dynamics through serotonin 5-HT2A receptor activation. Human neuroimaging studies have shown altered default mode network integrity, reduced network segregation, and broad changes in functional connectivity after psilocybin administration (Carhart-Harris et al., 2012; Madsen et al., 2021; Siegel et al., 2024; Ly et al., 2018). Preclinical studies further suggest that serotonergic psychedelics can promote structural and functional plasticity, including dendritic growth and synaptic remodeling (Vann Jones and O'Kelly, 2020).

Clinical investigation of psilocybin has focused primarily on psychiatric disorders and, more recently, on depression and anxiety in individuals with mild cognitive impairment or early Alzheimer’s disease. Published clinical data in advanced dementia remain extremely limited (Zheng et al., 2024). This report describes a case of transient multidomain functional improvement in advanced Alzheimer’s disease following high-dose psilocybin-containing mushroom administration.

## Case presentation

The patient was an octogenarian Japanese-American woman who lived with continuous family supervision and caregiver support. Progressive cognitive and functional decline had evolved over approximately 10 years. During the preceding 5 years, verbal output became predominantly monosyllabic, accompanied by severe reduction in spontaneous interaction, chronic urinary incontinence, executive dysfunction, impaired mobility, dysphagia, and marked dependence in activities of daily living.

### Clinical findings

Baseline examination revealed marked hypofunction, flat affect, reduced spontaneous verbal output, prolonged somnolence, impaired executive behavior, dependent ambulation, dysphagia, urinary incontinence, and severe reduction in social reciprocity and emotional expressiveness.

### Diagnostic assessment

The diagnosis of advanced Alzheimer’s disease had been established clinically approximately 10 years prior to intervention. The longitudinal progressive neurodegenerative course, profound episodic memory impairment, language reduction, executive dysfunction, and functional decline were considered clinically most compatible with advanced Alzheimer’s disease.

Although the clinical course was considered most consistent with advanced Alzheimer’s disease, formal biomarker confirmation and advanced neuroimaging were not available in the present real-world setting. Therefore, mixed or alternative neurodegenerative contributions, including vascular components, cannot be fully excluded. No evidence suggesting acute delirium, intoxication, or an alternative acute neurological diagnosis was identified during the observation period.

### Intervention

A single oral dose of 5 g of psilocybin-containing mushrooms (Enigma strain) was administered during the initial intervention. One month later, a second supervised session using 3 g of psilocybin-containing mushrooms was performed due to persistence of clinically meaningful improvements, including sustained urinary continence.

The intervention was exploratory and observational in nature, as no established psilocybin dosing framework currently exists for advanced dementia. The selected mushroom dose was relatively high compared with dosing approaches commonly used in modern clinical trials and was chosen based on prior experiential observations regarding depth and duration of psychedelic-induced neurobehavioral effects.

### Acute phase

The acute phase of the first session was characterized by clinically suspected hyperthermia, profuse sweating, profound somnolence, and a prolonged deep sleep-like state. Exact quantitative temperature measurements were not available. Approximately 19 h after administration, the patient spontaneously initiated autobiographical conversation lasting several hours.

During the second session, the patient remained significantly more verbally expressive throughout the experience and described emotionally positive imagery involving surfing with her son on a peaceful island. Facial expressivity, emotional reciprocity, spontaneous humor, and gait agility appeared markedly improved.

No severe persistent adverse effects, prolonged agitation, clinically significant cardiovascular instability, persistent psychotic symptoms, delayed neurological deterioration, or delayed medical complications were observed during follow-up.

### Follow-up and outcomes

Several clinically meaningful improvements persisted for weeks following the first intervention, including restoration of urinary continence, improved mobility, enhanced emotional reciprocity, increased spontaneous communication, and improved contextual social interaction (Table 1).

**Table 1:** Timeline of functional gains post-intervention.

Time post-intervention	Domain	Clinical observation
0–12 h	Autonomic/sleep	Clinically suspected hyperthermia, profuse sweating, prolonged deep sleep-like state
~19 h (03:30)	Language/autobiographical memory	Spontaneous awakening, ~4 h of autobiographical speech
Day 1	Alertness/social	Increased alertness, recognition of family
Day 2	Motor	Independent ambulation
Days 2–3	Executive/autonomy	Dressing self, *spontaneous initiative*
Days 2–3	Continence	Dry diapers, including nighttime
Days 6–7	Working memory/episodic	“*Where did Celso go?*”, correct vehicle recognition
Days 6–7	Attention/affective	*Sustained eye contact, reciprocal smiling*

Acute autonomic activation and a prolonged deep sleep-like state were followed by spontaneous autobiographical speech approximately 19 h post-administration and progressive recovery across motor, executive, continence, memory, and social-affective domains over subsequent days and weeks.

One month after the initial session, the patient remained continent and functionally improved compared with baseline. A second supervised psilocybin session using 3 g was subsequently performed and was associated with greater verbal expressivity, improved facial mimicry, spontaneous humor, emotionally valenced autobiographical imagery, and increased agility while walking.

The patient spontaneously stated: “It is pleasant to come here.”

### Patient perspective

Due to advanced cognitive impairment, formal structured self-report was limited. Nevertheless, during follow-up the patient spontaneously expressed emotionally positive statements regarding the sessions, including “It is pleasant to come here,” accompanied by increased emotional expressivity and autobiographical communication.

## Discussion

Observed gains were objective across autonomic, motor, executive, memory, affective, and social domains. The persistence of urinary continence after more than 5 years of chronic incontinence is particularly notable, given that continence depends on integrated interoceptive awareness, executive inhibition, and fronto-insular network function.

Recent human studies investigating psilocybin and sleep architecture have shown heterogeneous findings. In healthy volunteers, oral psilocybin increased REM sleep latency without major alterations in overall sleep–wake states (Dudysova et al., 2020). In contrast, clinical reports in depression have described improvements in sleep disturbances following psilocybin administration (Reid et al., 2024). The prolonged deep sleep-like state observed in the present patient may therefore reflect interactions between psilocybin-induced network modulation and altered baseline neurophysiology characteristic of advanced Alzheimer’s disease.

Beyond structural neuroplasticity, recent studies demonstrate marked reorganization of large-scale brain networks following psilocybin administration, including increased global integration, cortical desynchronization, and transient desegregation of canonical cortical systems (Madsen et al., 2021; Siegel et al., 2024; Ly et al., 2018; Silverstein et al., 2025). Such findings support the hypothesis that psilocybin may transiently facilitate functional reintegration of residual neural systems in neurodegenerative disease.

The present report has important limitations, including the single-case design, absence of formal polysomnographic monitoring, quantitative electrophysiology, neuroimaging biomarkers, and standardized cognitive scales. Causality cannot be established, and spontaneous fluctuations inherent to neurodegenerative disease cannot be completely excluded.

Importantly, mechanistic interpretations remain speculative. The present report should be understood primarily as a detailed observational description intended to generate hypotheses for future controlled investigation. The findings should not be interpreted as reversal of Alzheimer’s pathology. Rather, they raise the possibility that latent functional capacities may persist in advanced neurodegeneration and become temporarily accessible under specific neuromodulatory conditions.

## Conclusion

Residual functional capacity may persist in advanced Alzheimer’s disease and may become transiently accessible following psilocybin-induced modulation of large-scale brain networks. Systematic investigation is warranted.

## Data Availability

The original contributions presented in the study are included in the article. Further inquiries can be directed to the corresponding author.
